# Study of Cgrp-Receptors in Human Isolated Middle Meningeal Arteries Using Bibn4096Bs, Compound 1, and HαCgrp(8-37)

**DOI:** 10.1100/tsw.2001.437

**Published:** 2001-11-10

**Authors:** Z. Razzaque, D. Shaw, M. Bock, L. Maskell, J. Pickard, D. Sirinathsinghji, J. Longmore

**Affiliations:** ^1^ Merck Sharp & Dohme Research Laboratories, Terlings Park, Eastwick Road, Harlow, Essex, CM20 2QR, UK; ^2^ MRL, Chemistry Department, West Point, PA, USA; ^3^ Neurosurgery Unit, Addenbrookes Hospital, Hills Road, Cambridge, CB2 2QQ, UK

## Abstract

Calcitonin, CGRP, adrenomedullin, and amylin require both CRLR (calcitonin-gene receptor like receptor) and receptor activity modifying proteins (RAMP1, RAMP2, and RAMP3) in different combinations for expression of selective, functional receptors[1]. We investigated whether the antagonists BIBN4096BS[2], Compound 1 (WO98/11128, [3]), and CGRP(8-37) are functionally selective for CGRP receptors in human middle meningeal arteries (HMMA).

